# Antibody-drug conjugate therapies in multiple myeloma—what’s next on the horizon?

**DOI:** 10.37349/etat.2022.00067

**Published:** 2022-01-14

**Authors:** Monique Hartley-Brown, Paul Richardson

**Affiliations:** Department of Medicine, Division of Medical Oncology, Harvard Medical School, Dana-Farber Cancer Institute, Boston, MA 02215, USA; Institute of Oncology Research, Switzerland

**Keywords:** Antibody-drug conjugates, multiple myeloma, cancer, immunotherapy, targeted therapy

## Abstract

Targeted immunotherapy has arisen over the past decade to the forefront of cancer care. Notably, targeted therapies such as antibody-drug conjugates (ADCs) are becoming more recognized for a novel approach in cancer treatment. The mechanism of action of ADCs incorporates a monoclonal antibody portion directed against the tumor cell antigen and attached to the tumoricidal portion via chemical linkage. The binding of the monoclonal antibody portion allows for tumor cell internalization of the ADC and precise release of the toxic payload within the cancer cell. Multiple myeloma (MM) is an incurable cancer for which belantamab mafodotin was the first-in-class ADC to achieve United States Food and Drug Administration (FDA) approval for treatment of this disease. Clinical trials are currently evaluating other ADCs in the treatment of MM. In this review, a look at the current ADCs being tested in MM clinical trials with a focus on those that are more promising and a potential next-in-line for FDA approval for treatment of MM is discussed.

## Introduction

Multiple myeloma (MM) is an incurable plasma cell malignancy and the second most common adult hematologic malignancy. The disease is defined clinicopathologically by the (S)ixty percent plasmacytosis in the bone marrow, (Li)ght chain ratio (involved: uninvolved) above 100, (M)agnetic Resonance Imaging showing 1 or more focal myeloma lesion greater than 5 mm in size, (C)alcium elevation, (R)enal insufficiency due to myeloma, (A)nemia, and (B)one disease due to myeloma (SLiM-CRAB) criteria, per the International Myeloma Working Group (IMWG) [1]. MM is a very heterogeneous disease in which there is persistent innate and adaptive immune dysfunction [2]. The dysregulatory nature of the immune system within the disease lends itself well to the use of immunotherapies in MM.

Historically the treatment of MM involved the use of steroids and melphalan, an alkylating chemotherapeutic agent [3]. In the late 1990s, there was a breakthrough of novel therapies, namely immunomodulatory drugs (IMiDs) and proteosome inhibitors (PIs) [4]. The novelty of these therapies was noted in the mechanism of action for these types of therapeutics; wherein the target effects were mainly via specific cellular proteins that resulted in deleterious effects on the myeloma cells, as well as favorable effects within the bone marrow microenvironment [4]. Since then, there has been an emergence of more targeted immune therapeutics such that specific therapeutic drugs have a unique targeted protein or antigen on or within the myeloma cells that are crucial to the development of malignant clones. Targeted immunotherapy includes a plethora of drugs with a unique mechanism of action that has for the most part involved intelligent utilization of the immune system to directly target and attack cancer cells. In MM, these targeted therapies include monoclonal antibodies, antibody-drug conjugates (ADCs), bispecific and tri-specific antibodies, and chimeric antigen receptor T-cell (CAR-T) therapy, to name a few. Through targeting these antigens and triggering subsequent mechanisms of myeloma cell kill, for example via antibody-dependent cellular cytotoxicity (ADCC), antibody-dependent cellular phagocytosis (ADCP), cytokine-dependent cytotoxicity (CDC), or apoptotic effects. The development of these therapeutics has increasingly expanded over the past 5 years. Yet, despite advances with these medications, MM remains incurable.

**Figure 1.**
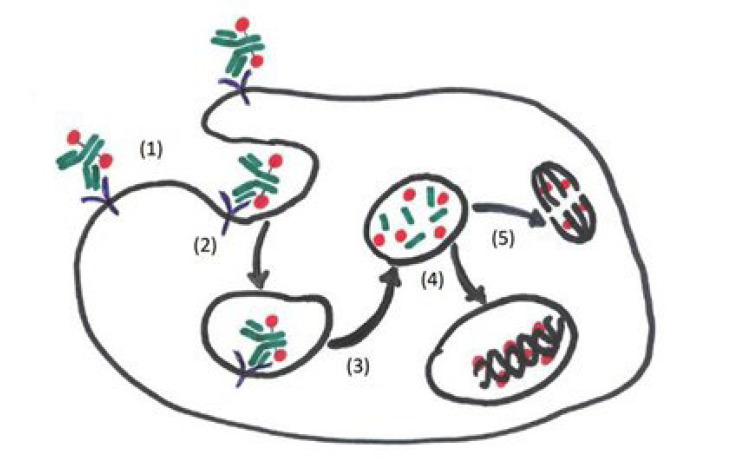
Mechanism of action of ADCs. (1) Circulating ADCs bind to target antigens on the myeloma cell via the monoclonal antibody portion; (2) the ADCs are endocytosed in endosomes; (3) ADCs are then degraded in lysosomes releasing the conjugated cytotoxic payload (toxin, chemotherapy agent, radioisotope, etc.); (4) the cytotoxic payload exerts (deoxyribonucleic acid) DNA damaging effects in the nucleus resulting in cell death; (5) the cytotoxic payload exerts its effects in the cytoplasm via microtubular polymerization and inhibition causing apoptosis

This review discusses ADCs that have been United States Food and Drug Administration (FDA)- approved and those that are promising in the progress of preclinical and clinical trial evaluation. Included in this review are ADCs, which are composed of monoclonal antibodies, directed to specific tumor antigens, chemically linked to a cytotoxic drug. Some of these antibody targets include B cell maturation antigen (BCMA), cluster of differentiation 38 (CD38), CD46, CD74, CD56, and CD138 [5]. The mechanism of action of ADCs is unique whereby the ADCs bind extracellularly via the antibody to the specific antigen, resulting in the tumor cell internalization of the ADCs. Once the ADCs are intracellular, lysosomal degradation occurs causing the release of the toxic payload within the tumor cells. The freed toxic payload enters the cytoplasm and/or the nucleus, exerting its effects and ultimately causing cytotoxic cell death (Figure 1). The advantages of ADCs unique mechanism of action include reduced toxicity to bystander normal tissues, increased tumor selectivity, and allows for use of highly potent cytocidal drugs that would otherwise be too toxic for sole systematic delivery [6, 7]. Disadvantages of ADCs include increased clearance rates and ADC aggregation resulting in reduced efficacy. Premature release of the toxic payload resulting in “bystander effects” on healthy cells, is another known disadvantage of ADCs.

The use of ADCs in MM has resulted in improved outcomes for patients in terms of improved measurable residual disease, and progression-free survival (PFS). ADCs have been shown to be efficacious in MM both in the preclinical and clinical settings. As such, one ADC has been FDA-approved for use in MM and several others are currently being evaluated in clinical trials.

## Belantamab mafodotin

Belantamab mafodotin (Bela) is the first FDA-approved BCMA targeted ADC [8]. BCMA is highly expressed on myeloma cells and mature B cells, and thus an ideal target antibody for MM. The normal function of BCMA binding on myeloma cells is to trigger downstream signaling intracellularly via the B-cell activating factor (BAFF) pathway, thus promoting plasma cell survival and proliferation [9]. Bela consists of a fully humanized immunoglobulin G1 (IgG1) antibody attached to the toxin, monomethyl auristatin F (MMAF) via a maleimidocaproyl linker. The parent IgG1 BCMA directed antibody exists in the afucosylated form, it then binds to the myeloma cell surface BCMA resulting in cellular internalization of the ADC. Once intracellular the MMAF, a tubulin polymerization inhibitor is released resulting in myeloma cell death [10, 11].

Myeloma cell death occurs through direct intracellular action of the released MMAF causing cell cycle arrest and apoptosis. Additionally, myeloma cell death occurs via enhanced binding to FcɣRIIIa receptors on the immune effector cell surface, due to the afucosylation of Bela, allowing for immune cell recruitment, ADCC and ADCP [11].

The first in human phase I trial with Bela was the DRiving Excellence in Approaches to MM 1 (DREAMM-1) study, evaluating Bela as a single agent in relapsed refractory MM (RRMM) patients. These patients were heavily pretreated with a median of 6 prior regimens, including prior PIs, IMiDs, and anti-CD38 monoclonal antibody exposure (daratumumab). At the one-year analysis, the overall response rate (ORR) was an impressive 60% [95% confidence interval (CI): 3.1-not estimable (NE)], with complete response (CR) rates of 15% and median duration of response (DoR) of 14.3 months. The daratumumab exposed/refractory patients also had a remarkable ORR of 38.5% (95% CI: 13.9–68.4) [12]. This led to the pivotal phase II DREAMM-2 clinical trial resulting in FDA approval of Bela.

The DREAMM-2 trial showed a very favorable ORR of 31% at a dosing of 2.5 mg/kg every 3 weeks. The patients in this trial also had RRMM and many were penta-refractory to IMiDs, PIs, and anti-CD38 therapies. The notable novel toxicity found with Bela is keratopathy, which affected 44% of patients but was manageable with delays in dosing and/or reduction of the dose at subsequent cycles [13]. The efficacy of Bela has been shown in subsequent combination trials to be substantial even at doses as low as 1.9 mg/kg and at delays of 6–8 weeks dosing per cycle [14, 15]. Currently, Bela is being evaluated in clinical trials in earlier lines of therapy and in combination with prior approved regimens such as PIs and IMiDs (Table 1). Examples of such ongoing phase III clinical trials include DREAMM-8, evaluating Bela in combination with pomalidomide (Pom) and dexamethasone (Dex) as second-line therapy; DREAMM-9 evaluating Bela with bortezomib (Bor), lenalidomide (Len), and Dex in newly diagnosed MM (NDMM) [7, 9, 16]. Phase II trials involving Bela combinations include DREAMM-4 evaluating Bela with pembrolizumab in RRMM [17]; DREAMM-5, evaluating Bela in combination with several other agents in the fourth-line setting RRMM [18], and DREAMM-6 comparing Bela in combination with Len and Dex *versus* Bela with Bor and Dex in RRMM [19]. The efficacy, favorable ORR, and PFS, along with the manageable toxicity profile of Bela in patients with RRMM have opened the doorway for the application of other ADCs in the preclinical and clinical trial settings.

**Table 1.** ADCs in MM

**Target**	**Agent**	**Active key trials (phase)**	**Prior lines**	**Regimen**	**Toxicities (≥ grade 3)**
BCMA	Belantamab mafodotin	NCT03715478 (Ph I/II)	RRMM	+ Pom/Dex	Keratopathy/MEC, ↓ VA, neutropenia, thrombocytopenia, lung infection, dyspnea
		NCT03848845 DREAMM-4 (Ph II)	RRMM	+ Pembrolizumab	-
		NCT04126200 DREAMM-5 (Ph II)	3 or more	+/− GSK3174998 or +/− GSK3359609 or +/− nirogacestat or +/− dostarlimab	-
		NCT03544281 DREAMM-6 (Ph II)	RRMM	+ Len/Dex or + Bor/Dex	Keratopathy/MEC, thrombocytopenia
		NCT04484623 DREAMM-8 (Ph III)	1 or more	+ Pom/Dex	-
		NCT04091126 DREAMM-9 (Ph III)	TI-NDMM	+/− Bor/Len/Dex	-
		NCT04398745 DREAMM-12 (Ph I)	2 or more	Single agent	-
		NCT04177823 (Ph I)	2 or more	Single agent	-
	MEDI2228	NCT03489525	RRMM	Single agent	Thrombocytopenia, pleural effusion, rash, transaminitis
	AMG 224	NCT02561962 (Ph I)	3 or more	Single agent	Thrombocytopenia, transaminitis, fatigue, nausea, anemia
CD38 (ADC)	TAK-169	NCT04017130 (Ph I)	2 or more	Single agent	-
	TAK-573	NCT03215030 (Ph I/II)	3 or more	+/− Dex	-
CD46 (ADC)	FOR46	NCT03650491 (Ph I)	RRMM	Single agent	-
CD74 (ADC)	STRO-001	NCT03424603 (Ph I)	RRMM	Single agent	-

↓: decreased; -: not applicable; Ph: clinical trial phase; TI-NDMM: transplant-ineligible newly diagnosed MM; MEC: microcyst- like epithelial changes; VA: visual acuity

## MEDI2228

MEDI2228, another BCMA targeted ADC, conjugated to the toxin pyrrolobenzodiazepine (PBD) tesirine, appeared initially promising. Cellular internalization of the drug via BCMA extracellular bonding allows for intracellular lysosomal cleavage of the PBD portion resulting in downstream DNA crosslinking and apoptotic myeloma cell death [20, 21]. The phase I trial analysis reported results of an initial 82 patients, showed an ORR of 65.9% (95% CI: 49.4–79.9). Patients were heavily pretreated, between 2–11 lines of previous therapy, with 23/41 patients in the MEDI2228 treatment arm triple refractory to IMiDs, PIs, and anti-CD38 therapy. Ocular toxicity, but not keratopathy as well as serositis reflected by pleural effusions was experienced in patients, unlike that seen in Bela cases. The agent was poised for phase II trials in 2021, however, it was withdrawn by the company for other safety and efficacy concerns [22].

## AMG 224

AMG 224 is composed of a humanized IgG1 anti-BCMA antibody conjugated to mertansine, a microtubule inhibitor, attached to the non-cleavable linker 4-(*N*-maleimidomethyl) cyclohexane-1-carboxylate [23]. Published reports from the first in human phase I trial with AMG 224 showed data on 42 patients. Patients were heavily pretreated with a median of 7 prior lines of therapy. The maximum tolerated dose (MTD) found was 190 mg administered every 3 weeks. The grade 3 or higher adverse drug effects (ADEs) were thrombocytopenia (55%), neutropenia (27%), and anemia (18%). The ocular effects requiring treatment were all grade 1–2. There were no fatal adverse events (AEs) in this study. The ORR was 23% (40 evaluable patients; 95% CI: 11–39%), including 2 stringent CRs, 2 very good partial responses (VGPRs) and 5 partial responses (PRs). The median DoR was 14.7 months, with the 2 stringent CRs obtaining the DoRs 29.8 and 19.2 months [23]. The favorable toxicity profile and notable efficacy of this agent in such heavily pretreated RRMM patients illustrated in this initial phase I trial with AMG 224 indicates its potential for further assessment in ongoing clinical trials.

## TAK-169

TAK-169 is conjugated to Shiga-like toxin A subunit (SLTA) with the antibody portion targeting CD38. The SLTA, on cellular entry, undergoes intracellular enzymatic and irreversible inhibition of protein synthesis. Preclinical studies suggest this agent would be effective in daratumumab refractory/exposed RRMM patients. The data for this agent remains immature, with early phase I trials in the process to assess the future clinical potential for TAK-169 in the clinical setting [24] (Table 1).

## TAK-573

Phase I/II clinical trial data results for TAK-573 have shown promise. The TAK-573 antibody portion targets CD38 and is conjugated to an attenuated interferon-alpha 2b as the toxin payload. A recent study, by Vogl et al. [25], showed data of 59 RRMM patients. Patients were heavily pretreated, 93% daratumumab exposed and 14% CAR-T therapy exposed. The median was 7 lines prior therapy. The MTD was not reached. Safety and efficacy were tolerable with grade 3 and higher toxicities being thrombocytopenia and neutropenia, respectively. The recommended phase 2 dose (RP2D) and appropriate infusional dosing have yet to be determined [25]. This is another ADC agent that shows significant potential in the myeloma treatment space (Table 1).

## FOR46

FOR46 antibody portion targets the myeloma cell surface antigen CD46. The anti-CD46 antibody is conjugated to the toxic payload MMAF. This agent has a unique effect which is ideal in the setting of gain of long arm (q) of chromosome 1 (chromosome *1q*) pathology. The patients with gain of chromosome *1q* overexpress CD46 on the malignant myeloma cells making them more sensitive to this drug [26]. The initial phase I clinical trial of FOR46 began in early 2020 and is ongoing (Table 1). Preliminary results have not yet been published.

## STRO-001

STRO-001, like milatuzumab (IMMU-115; an ADC no longer in clinical trials due to failed responses in the phase I trial) targets the aglycosylated IgG specific CD74 but is conjugated to the toxin maytansinoid. The CD74 transmembrane protein is highly expressed on myeloma cells and macrophages, notably less expression on other cell lines. Preclinical study results in myeloma cell lines and mice showed encouraging results for myeloma cell kill [27]. The first-in-human phase I trial (NCT03424603) evaluated 14 RRMM heavily pretreated patients (median number of 6 prior therapies), revealed safety and efficacy as monotherapy. The most common AEs are low-grade (grades 1–2) chills, fatigue, fever, nausea, cough, and infusion reactions. The MTD was not reached with the highest dose tested being 0.91 mg/kg and 2 dose-limiting toxicities (DLTs) of thromboembolic events [28] (Table 1).

## HDP-101

HDP-101, another BCMA targeted ADC, is conjugated to the toxic payload alpha-amanitin [29]. The novelty of HDP-101 is its preferential efficacy in deletion *17p* pathology. The alpha-amanitin toxin preferably binds RNA polymerase II (POLR2A), like tumor protein p53 (TP53)-the tumor suppressor gene affected in deletion *17p* patients, is located on the short arm of chromosome 17. These two, TP53 and POLR2A are in proximity, thus in patients with deletion *17p*, both are often co-deleted. This uniquely allows HDP-101 to be more effective in the deletion *17p* pathology. The first-in-human phase I/II trial of this agent is ongoing, as yet early published data is unavailable for review [30]. Preclinical data shows hepatotoxicity with this agent. Additionally, preclinical reports have shown the efficacy of this agent is not only proliferating myeloma cells but also dormant malignant myeloma cells, suggesting a deeper response with this agent in clearing the myeloma cell niche [30, 31]. Preclinical evaluation has shown intravenous and subcutaneous administration of this agent to be effective in myeloma cell kill with cell lines refractory to 3 and 4 lines of prior therapy [30, 31].

## Indatuximab ravtansine

The antigen target for indatuximab ravtansine (BT062) is CD138, a transmembrane protein receptor known to be overexpressed in malignant myeloma cells. The BT062 anti-CD138 chimeric antibody is conjugated to the toxin ravtansine (DM4). Ravtansine is a microtubule-binding cytotoxin, also known as maytansinoid DM4, which once internalized within the myeloma cells releases DM4 post lysosomal degradation of its linker. The free DM4 binds tubulin subsequently causing cell cycle arrest and apoptosis of the myeloma cells [32].

Reports from the first-in-human phase I trial with 32 patients, showed the MTD to be 160 mg/m^2^ with every 3-weekly dosing. A second phase I/IIa trial included 35 patients scheduled on a 28-day cycle, with BT062 given once weekly for 3 weeks on and 1 week off, resulting in the RP2D of 140 mg/m^2^, with a median overall survival (OS) of 26.7 months [33].

Evaluation of BT062 with IMiDs, specifically, Pom or Len and Dex have also been done. The multi-center open-label phase I/IIa trial results were reported earlier in 2021. The Len with BT062 and Dex cohort had failed at least one prior therapy, whereas the Pom, BT062, and Dex cohort had failed at least two prior therapies, and had evidence of disease progression within 2 months of the last therapy. There were 64 evaluable patients, 47 (73%) were allocated to the Len cohort, and 17 (27%) to the Pom cohort, with a median follow-up of 24.2 months (95% CI: 19.9–45.4) and 24.1 months (95% CI: 17.7–36.7), respectively. Results showed the MTD of BT062 in the Len group to be 100 mg/m^2^; this was used as the RP2D for the Pom group. The ORR for the Len *versus* Pom group was 71.7% (33/46 patients) and 70.6% (12/17 patients), respectively. The clinical benefit response (CBR; defined as ORR + minor response) for the Len and Pom groups was 85% (39/46 patients) *versus* 88% (15/17 patients), respectively. Notable AEs equal or beyond grade 3 were neutropenia, anemia, and thrombocytopenia, with 55% (35/66 patients) requiring treatment discontinuation. There were 8% (5 patients) with AEs resulting in a fatality; however, none of the fatal events were reported as related to the BT062 [33]. These initial results indicate tolerability and efficacy of BT062 both as a single agent and in combination with Len or Pom and Dex. Further studies to evaluate its clinical applicability are underway and will potentially add a new agent to the arsenal of ADC therapies in the treatment of MM.

## Lorvotuzumab mertansine

Lorvotuzumab mertansine (IMGN901), like BT062, has a maytansinoid toxic payload, in this case, DM1, also known as maytansinoid mertansine. The antibody component is a humanized antibody that targets CD56. The first-in-human phase I trial with lorvotuzumab mertansine, reported treatment of 37 patients, 42.9% achieving equal to or better than stable disease (SD), with an MTD of 112 mg/m^2^ and a median DoR of 15.5 months. AEs of grade 3 and higher were fatigue and reversible renal insufficiency [34]. The combination phase II trial with Len and Dex showed an ORR of 56.4% at the 75 mg/m^2^ lorvotuzumab dose. The toxicity profile was manageable, with neuropathy being most common but less than grade 3 [35]. These results are encouraging for further clinical investigation with this agent.

## IMMU-115

This agent targets CD74 and is conjugated to doxorubicin, though initially with promising preclinical data was no longer evaluated after the phase I single-agent study revealed no significant clinical responses [36, 37].

## CC99712

CC99712 targets the BCMA antigen on myeloma cells, with monomethyl auristatin E (MMAE) as the toxic payload. Preclinical trials are underway with this agent. Initial phase I trial results of safety, tolerability, and MTD have yet to be published, as trials are ongoing. This may potentially be another ADC agent used for treating RRMM in the near future [38].

## Conclusions

ADCs are well-engineered targeted therapeutics that have a unique mechanism of action allowing for more favorable cancer cell kill. These modern immunotherapies are being increasingly developed for the treatment of many malignancies including MM. There is now evidence of clinical benefit from this drug-class in MM and in particular with belantamab mafodotin, which will likely translate into real-world practice, and advance the survival outcomes of patients with this disease, not least because of the “off the shelf” basis to their use [39]. These are examples of some of the current ADCs in clinical development for the treatment of MM, and advancing the survival outcomes of patients with this disease. Many of the candidate drugs still need to be developed beyond the preclinical and phase I clinical settings. There are, however, promising clinical trials for MM patients currently and on the horizon. The hope is as these therapeutic advances develop and mature, with improved efficacy especially in combination, the field will move closer to a functional cure in an increasing proportion of MM patients.

## Abbreviations

ADCs: antibody-drug conjugates
AEs: adverse events
BCMA: B cell maturation antigen
Bela: belantamab mafodotin
Bor: bortezomib
BT062: indatuximab ravtansine
CD: cluster of differentiation
CI: confidence interval
CR: complete response
Dex: dexamethasone
DM4: ravtansine
DoR: duration of response
FDA: Food and Drug Administration
IgG1: immunoglobulin G1
IMiDs: immunomodulatory drugs
Len: lenalidomide
MM: multiple myeloma
MMAF: monomethyl auristatin F
MTD: maximum tolerated dose
ORR: overall response rate
PIs: proteosome inhibitors
Pom: pomalidomide
RP2D: recommended phase 2 dose
RRMM: relapsed refractory multiple myeloma

## Declarations

### Copyright

© The Author(s) 2022.
